# Crop Classification in Satellite Images through Probabilistic Segmentation Based on Multiple Sources †

**DOI:** 10.3390/s17061373

**Published:** 2017-06-13

**Authors:** Oscar S. Dalmau, Teresa E. Alarcón, Francisco E. Oliva

**Affiliations:** 1 Centro de Investigación en Matemat́icas, CIMAT, 36023 Guanajuato, Mexico; 2 Centro Universitario de los Valles, Universidad de Guadalajara, 46600 Ameca, Jalisco, Mexico; francisco.oliva@profesores.valles.udg.mx

**Keywords:** probabilistic segmentation, remote sensing, likelihood, vegetation indices, histogram

## Abstract

Classification methods based on Gaussian Markov Measure Field Models and other probabilistic approaches have to face the problem of construction of the likelihood. Typically, in these methods, the likelihood is computed from 1D or 3D histograms. However, when the number of information sources grows, as in the case of satellite images, the histogram construction becomes more difficult due to the high dimensionality of the feature space. In this work, we propose a generalization of Gaussian Markov Measure Field Models and provide a probabilistic segmentation scheme, which fuses multiple information sources for image segmentation. In particular, we apply the general model to classify types of crops in satellite images. The proposed method allows us to combine several feature spaces. For this purpose, the method requires prior information for building a 3D histogram for each considered feature space. Based on previous histograms, we can compute the likelihood of each site of the image to belong to a class. The computed likelihoods are the main input of the proposed algorithm and are combined in the proposed model using a contrast criteria. Different feature spaces are analyzed, among them are 6 spectral bands from LANDSAT 5 TM, 3 principal components from PCA on 6 spectral bands and 3 principal components from PCA applied on 10 vegetation indices. The proposed algorithm was applied to a real image and obtained excellent results in comparison to different classification algorithms used in crop classification.

## 1. Introduction

The segmentation process allows us to divide the image into significant parts according a certain criterion. Clustering algorithms such as k-means, fuzzy c-means and the Iterative Self-Organizing Data Analysis Technique (ISODATA) algorithm [1] have been used successfully for segmentation problems, however these methods per se, do not consider the contextual information for a pixel, what is necessary to obtain a good segmentation. A very effective approach for including the features of the pixel neighborhood is the Bayesian estimation together with the Markov Random Field (MRF), [2,3,4,5,6,7,8,9]. With this approach, a label field is computed assuming that a dependence exists between all probability distributions of the pixels belonging to the same neighborhood. This assumption is specified by considering a Markovian priori distribution. Gauss Markov Measure Field (GMMF) [4] is one of the models that combines Bayesian estimation with Markov Random Field and it is used in many classification tasks, [5,6,7,8,10,11,12,13]. One of the main difficulties for GMMF, as for all methods based on the combination of Bayesian estimation and MRF for image segmentation, is the likelihood computation. In the case of 1D or 3D feature spaces the likelihood can be computed based on the corresponding normalized histograms. However, the computation of the likelihood becomes a very hard problem when the number of features increases. In the case of crops classification for satellite images the number of features is high, which is a limitation for the direct use of the GMMF [4]. On the other hand, crops classification is a complex task due to the similarity of the spectral signatures among different crops. Hence, the selection of the feature space, i.e., information sources, is a key step in this research, so that we can use the GMMF as a classifier that allows us to incorporate contextual information and to reach good classification results.

In [14] authors considered a pixel based image approach in order to segment 5 different land cover types in Russia. The experimental work included the minimum euclidean distance, the box classifier, Mahalanobis distance, the maximum likelihood classifier and clustering techniques. The feature space was composed of blue, green and red bands. The best performance was achieved by the maximum likelihood. Authors in [15] used three different vegetation indices: the Normalized Difference Vegetation Index (NDVI) [16], the Green Normalized Difference Vegetation Index (GNDVI) [17], and the Normalized Difference Red Edge Index (NDRE) [18] for crop classification in the region located in Turkey. All indices were computed taking into consideration the spectral bands obtained from the RapidEye satellite, which is the first high-resolution multispectral satellite system incorporating the red-edge band which is sensitive to vegetation chlorophyll [15]. Four different feature spaces were studied in the research in [15]: the first spectral space contains NDVI, GNDVI and NDRE indices, the others three feature spectral spaces are composed of only two of the three indices included in the first space. For crop classification, the support vector machine method [19] was used. The experimental work in [15] demonstrated that the spectral index NDRE provided the highest contribution to the support vector machine classifier and that the space composed of three spectral indices outperformed the rest of studied feature spaces. The proposal in [20] used ASTER data and studied different feature spaces in order to classify the sugarcane vegetation in Uttarkhand, India. The analyzed spaces were: feature space determined by the three bands located in the VNIR (visible and near infrared) region of the spectrum; and a combination between VNIR bands and NDVI transformation. The classifier used in this work was the maximum likelihood method and achieved the highest classifier precision using the combination of VNIR bands and NDVI index. The main drawback in [20] was the similarity of spectral signatures among sugarcane vegetation classes. The methodology described in [21] utilizes the green, red, near and short wave infrared bands from SPOT-5 satellite in order to identify crops in Texas. With the SPOT-5 data, the authors studied the performance of 5 classifiers: maximum euclidean distance, Mahalanobis distance, maximum likelihood, spectral angle mapper [22] and SVM [19]. The experimental work proved that the SPOT-5 data in conjunction with maximum likelihood and SVM allows to estimate crop areas. Researchers in [23] carried out a performance analysis of the following supervised classifiers: maximum likelihood, parallelepiped and Mahalanobis distance. As in [21] the feature space was built from SPOT-5 data. The goal of the research was the identification of the different land classes in Barcelona, Spain. Maximum likelihood outperformed other classifiers for almost all the objects present in the study image, however for water bodies the Mahalanobis distance leaded to best accuracy results. The selection of the spectral bands and the right classifier was a critical step [24,25,26]. Unlike the previous works, which are pixel-based, in [27] the researchers elaborated an object based image algorithm. The purpose of the algorithm was to identify 13 crop types in California, USA. The spectral information was taken from ASTER satellite, during three different growing-seasons periods. The proposal in [27] combines an object based approach with the decision tree rules. The feature space is composed of several vegetation indices derived from the visible, near and short waves bands, together with textural features extracted from the spectral bands. The experimental work evidenced that the textural features improved the discrimination among heterogeneous permanent crops. In this research also is emphasized the importance of NIR and SWIR bands for crop identification and it is studied the contribution of each feature to the classification accuracy. All the reported works, either pixel based or object based approaches face the same challenges in best discriminating a class: how do we select the appropriate feature space and how do we select the right classifier?

This work is an extension of our conference paper [28] and we propose a supervised approach for crop classification, in which multiple sources of information or features spaces can be combined, including contextual information. To this end, we propose a generalization of the GMMF model that allows us the combination of multiple sources of information and also considers the spatial or contextual information.

The structure of this paper is as follows: Section 2 details about the previous works, Section 3 explains the proposal, Section 4 is dedicated to the description and discussion of the experimental work and finally Section 5 contains our conclusions.

## 2. Previous Works

In Ref. [29], a supervised algorithm for crop classification in satellite images was proposed. The algorithm includes a segmentation step achieved through the GMMF model [4], in which the computation of the likelihood is an important ingredient. Equation (1) represents the GMMF approach proposed in [4].

(1) $$\begin{array}{ccc} p^{*} & = & \arg\min_{p}\sum_{r\in L}\sum_{k\in K}{\left(p_{k}\left(r\right)-v_{k}\left(r\right)\right)}^{2}+\lambda \sum_{s\in N_{r}}{\left(p_{k}\left(r\right)-p_{k}\left(s\right)\right)}^{2}, \end{array}$$

where $v_{k}\left(r\right)$ is the likelihood of pixel *r* to belong to the class *k*, λ>0 is a regularization parameter, $N_{r}$ represents a set of neighboring pixels to the pixel *r* and $p^{*}$ is the estimated probability distribution field, in this way $p^{*}\left(r\right)$ allows us to classify the pixel *r* by maximizing $p_{k}^{*}\left(r\right)$ over *k*. The solution of the optimization problem (1) can be obtained by the following Gauss-Seidel scheme:

(2) $$p_{k}\left(r\right)=\frac{v_{k}\left(r\right)+\lambda \sum_{s\in N_{r}}p_{k}\left(s\right)}{1+\lambda |N_{r}|}.$$

The final segmentation is obtained by using ’the winner takes it all’ strategy, i.e., given the vector field $p^{*}$, Equation (1), the segmentation is computed with the following equation:

(3) $$s\left(r\right)=\arg\max_{k\in K}p_{k}\left(r\right),\;\forall r\in L.$$

Authors in [29] estimated the likelihood by means of 3D histograms in the feature space composed of green, red and near infrared bands. It is well known that the information contained in these three bands allows to recognize crop patterns, [30]. The histograms were computed based on expert information.

**Figure 1:**
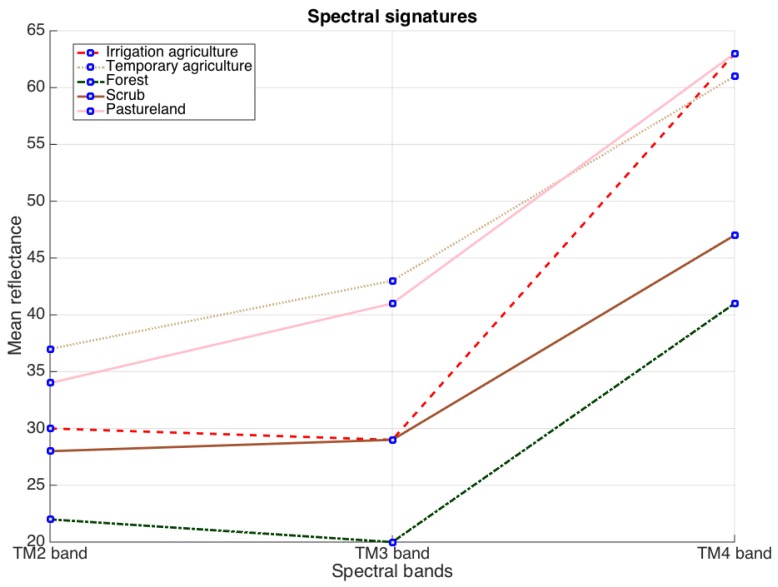
Mean reflectance values for the TM432 bands for each vegetation type under study.

Figure 1 represents the spectral signature of five crops studied in [28,29]. Observe the similarity of the response among all crops. This fact explains the precision errors of the proposed algorithm in [29]. For addressing this problem the authors in [28] considered using two different feature spaces (sources of information): the feature space composed of green, red and near infrared bands and the space based on the three principal components of 10 vegetation indices [15,16,31,32,33,34,35,36,37]. From these two feature spaces they constructed histograms, and from these histograms they estimated the corresponding likelihood. Different from [29], authors in [28] introduced a modification in data term of the GMMF approach in order to select at each pixel *r* the likelihood with the least entropy:

(4) $$\begin{array}{ccc} p^{*} & = & \arg\min_{p}\sum_{r\in L}\sum_{k\in K}\sum_{i=1}^{2}\omega_{i}\left(r\right){\left(p_{k}\left(r\right)-v_{k}^{i}\left(r\right)\right)}^{2}+\lambda \sum_{s\in N_{r}}{\left(p_{k}\left(r\right)-p_{k}\left(s\right)\right)}^{2}, \end{array}$$

where $v^{i}\left(r\right)$ is the likelihood that comes from the *i*-th source and the weight function $\omega_{i}\left(r\right)\in \left\{0,1\right\}$ is given by Equation (5)

(5) $$\begin{array}{ccc} \omega_{i}\left(r\right) & = & \left\{\begin{array}{cc} 1 & \mathrm{if}\;E\left(v^{i}\left(r\right)\right)<E\left(v^{3-i}\left(r\right)\right)\\ 0 & \mathrm{otherwise}, \end{array}\right. \end{array}$$

i∈{1,2} and E(·) is an entropy measure [38]. The authors in [28] used Gini impurity index, i.e.,

(6) $$\begin{array}{ccc} E(f) & = & 1-f^{T}f, \end{array}$$

such that $1^{T}f=1,f⪰0$.

From Equation (5) it is understandable that only the likelihood that leads to a probability distribution with a lowest entropy is included in the data term for the corresponding pixel, i.e., once the likelihoods for the two feature spaces are computed, the weight function (5) selects only one likelihood for each pixel. The previous idea [28] contributed to diminish the misclassification in the recognition of different crops. The inclusion of a new feature space improved the classification process with respect to the results obtained in [29]. Note that in practice, the functional in (4) selects only one feature space at each pixel. In this work we present an extension of our researches in [28,29] and we propose a generalization of the GMMF model that allows us the fusion of multiple sources of information for classification tasks. We evaluate the behavior of the proposal for crop recognition task using satellite images, however the proposal can be applied to other classification problems.

## 3. The Proposal

**Figure 2:**
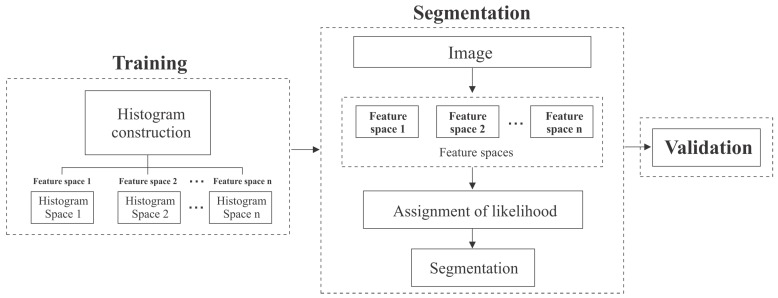
Stages of the proposed algorithm.

The segmentation approach is composed of three stages: training, segmentation and validation. In Figure 2 are illustrated the stages of the proposal. In the next sections we explain each stage.

### 3.1. Training Stage

The training stage is based on histograms construction [28,29]. We assume that the information about the classes has been provided by an expert, for example, by making scribbles on training images for each class. Let us denote the normalized histogram as:

(7) $$\begin{array}{ccc} h(x;k) & \propto & N(x;k), \end{array}$$

(8) $$\begin{array}{ccc} \sum_{x}h\left(x;k\right) & = & 1,\;\forall k\in K, \end{array}$$

where K={1,2,⋯,K} and *K* is the number of classes, x corresponds to a feature vector. For example, if one considers three spectral bands for satellite images; h(x;k) is a 3D histogram corresponding to the class *k*. The computed histograms allows us to estimate the likelihood of pixels of an image to belong to a class with respect to a feature space. This is an important step, because the computation of the likelihood is a required element for Markov Measure Field segmentation models [5,6,7,28,29,39,40].

Authors in Ref. [28] considered the use of only two feature spaces, now we propose to use multiple feature spaces or sources of information. Let us denote the number of feature spaces as $N_{f}$. Then, we build the normalized histograms $h^{f}\left(\cdot ,k\right)$ for each feature space $f\in F=\{1,2,\cdots ,N_{f}\}$ and class k∈K. Let us define the mapping function $x^{f}:L\subset Z^{2}\to R^{\left|f\right|}$ that allows us to extract for each site r∈L of an image the feature vector $x^{f}\left(r\right)\in R^{\left|f\right|}$ where |f| represents the size of the feature space *f*. Therefore, $h^{f}\left(x^{f}\left(r\right),k\right)$ can be interpreted as the probability of the feature vector $x^{f}\left(r\right)$ associated to pixel *r* given the class *k* and the feature space *f*.

### 3.2. Segmentation Stage through Multiples Sources and Probabilistic Approach

Here we assume that we have already trained our algorithm, i.e., we know the histograms $h^{f}\left(\cdot ,k\right)$ for each feature space *f* and for class *k*.

The segmentation procedure is as follows:

1. For a given image, we compute the feature spaces provided by the mapping $x^{f}\left(r\right),\;\forall r\in L$, f∈F.
2. Then, the likelihood is assigned according to the following equation
  (9) $$v_{k}^{f}\left(r\right)\propto h^{f}\left(x^{f}\left(r\right);k\right)$$
  such that $\sum_{k\in K}v_{k}^{f}\left(r\right)=1$, ∀r∈L, f∈F. The likelihood is obtained by normalizing $h^{f}\left(x^{f}\left(r\right);k\right)$ with respect to the classes.
3. Here we propose a robust GMMF approach that generalizes the proposal in [28] including more feature spaces and with weight functions in both the data and regularization terms. Equations (10)–(12) indicate the modifications.
  (10) $$\begin{array}{ccc} p^{*} & = & \arg\min_{p}\sum_{r\in L}\sum_{k\in K}\sum_{f\in F}\omega_{f}\left(r\right){\left(p_{k}\left(r\right)-v_{k}^{f}\left(r\right)\right)}^{2}+\lambda \sum_{s\in N_{r}}\omega_{rs}{\left(p_{k}\left(r\right)-p_{k}\left(s\right)\right)}^{2}, \end{array}$$
  (11) $$\omega_{f}\left(r\right)=\frac{\omega_{f}^{s}\left(r\right)}{\sum_{f\in F}\omega_{f}^{s}\left(r\right)}$$
  (12) $$\omega_{f}^{s}\left(r\right)=\frac{\mu }{\mu +U_{f}\left(r\right)}$$
  where $U_{f}\left(r\right)\in \left[0,1\right]$ is an uncertainty measure of the information source *f* at pixel *r*, for example, the measure of entropy (5) used in [28]; λ>0 is the regularization parameter, μ>0 controls the relative importance of the likelihood for different sources. When μ is very large, the contribution of the likelihood for all sources tends to be the same and when it is close to zero, the functional in (10) tends to select the likelihood corresponding to the lowest uncertainty, i.e., this tends to the solution proposed in [28] when using the entropy as uncertainty measure. The weight $\omega_{rs}\in \left[0,1\right]$ function allows to control the edges between classes, here we use
  (13) $$\begin{array}{ccc} \omega_{rs} & = & \frac{\mu }{{\mu +∥u\left(r\right)-u\left(s\right)∥}_{2}^{2}} \end{array}$$
  (14) $$\begin{array}{ccc} u_{k}\left(r\right) & \overset{def}{=} & \sum_{f\in F}\omega_{f}\left(r\right)v_{k}^{f}\left(r\right) \end{array}$$
  such that $\omega_{rs}\approx 1$ if the sites r,s very probably belong to the same class and $\omega_{rs}\approx 0$ otherwise. The solution of the optimization problem (10) yields the following Gauss-Seidel scheme:
  (15) $$p_{k}\left(r\right)=\frac{u_{k}\left(r\right)+\lambda \sum_{s\in N_{r}}\omega_{rs}p_{k}\left(s\right)}{1+\lambda \sum_{s\in N_{r}}\omega_{rs}}.$$
  which is similar to the Gauss-Seidel scheme (2), but now the term $u_{k}\left(r\right)$, Equation (14), is a mixture term that allows us to combine or fuse different likelihoods. In addition, the above formula also includes function weights to control the edges between classes. Note that, the first term in the numerator of the Equation (15) is a convex linear combination of likelihoods derived from different sources. Feature spaces with lower uncertainty have a greater $\omega_{f}\left(r\right)$ and therefore they have a greater contribution to the data term in Equation (10).

### 3.3. Validation

For validation purposes we use the measure of agreement Cohen’s kappa [41], the precision by class and the overall accuracy, see Section 4.4 for details. These comparison measures use the information derived from the confusion matrix [42,43]. This matrix is built through the manual segmentation given by an expert (ground truth) and the results obtained by the algorithms.

## 4. Experiments and Discussion

In this section we describe the experimental work and analyze the results obtained by our proposal when applied to crop classification task in satellite images.

### 4.1. Study Area

The study area is located in western Mexico at coordinates Lat. $20{}^{{}^{\circ}}39^{\prime }58^{\prime \prime }$ N, Long. $103{}^{{}^{\circ}}21^{\prime }7^{\prime \prime }$ W, with an altitude of 1550 m above sea level, in the Atemajac Valley [44]. We study five types of vegetation located in this region, see Table 1.

### 4.2. Data Sources

Data used in the experimental work are from Landsat-5 Thematic Mapper (TM) satellite imagery. The spectral band information for this satellite appears in Table 2.

The studied image has resolution of 30 m and $2^{8}$ radiometric resolution and corresponds to 1 March 2011. Data was delivered in level 1T, in which geometric correction was applied [45].

**Figure 3:**
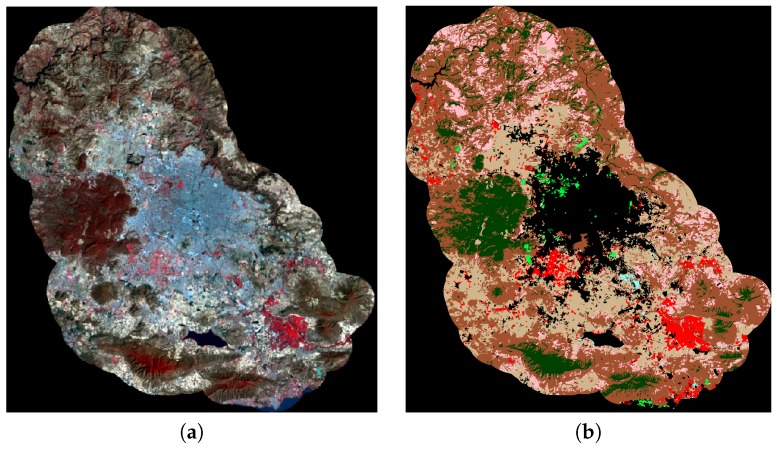
(**a**) Studied image; (**b**) ground truth given by an expert.

The image was obtained through the USGS Global Visualization Viewer site [46]. For error analysis, the image was manually segmented by experts Oliva et al. [28]. The studied image and the ground truth appear in Figure 3.

### 4.3. Studied Feature Spaces

Three feature spaces were analyzed in our work:

1. **Space 1**: it is composed of information from TM2, TM3 and TM4 bands due to it is well known [30] that these three bands contain relevant data for crop detection. The selected bands are preprocessed through a bilateral filter [47].
2. **Space 2**: it contains the first three principal components from the PCA applied on 10 vegetation indices, see Table 3. Such indices are based on mathematical operations on spectral bands and they allow to enhance the information related to vegetation. In order to compute the indices we calculate the reflectance values, ρ, corresponding to the acquired images, using the algorithms in [48], see also the procedures given in [49,50,51,52]. The included indices appear in Table 3. Symbols $\rho_{r}$, $\rho_{g}$, $\rho_{b}$ and $\rho_{NIR}$ denote the reflectance values for the red, blue, green and infrared bands respectively. We followed the recommendation given in [35], and set L=0.5 to compute the SAVI and SARVI expressions. For SARVI we considered γ=1 as authors in [34].
3. **Space 3**: this space contains three principal components from PCA applied on the spectral bands TM1, TM2, TM3, TM4, TM5 and TM7, see Table 2. Although TM2, TM3 and TM4 bands more accurately describes information related to vegetation [30], in this investigation PCA on all six mentioned bands is applied in order to include information from other spectral regions.

### 4.4. Comparison Measures

The comparison measures, for validation purposes, are based on the confusion matrix *C* which is used in multi-class classification problems [42,43] to assess the performance of algorithms. Here, we assume that the rows of the confusion matrix correspond to the actual classes (the “ground truth”) and the columns correspond to the predicted classes. Then, the entry $C_{ij}$ of the confusion matrix *C* represents the number of data of the class *i* that are predicted in class *j*. The performance measure we used for comparing the algorithms are: the overall accuracy, the precision for each class *j* and the Cohen’s kappa, i.e.,

(16) $$\begin{array}{ccc} \mathrm{Overall}\;\mathrm{accuracy} & = & \frac{\sum_{j=1}^{K}C_{jj}}{\sum_{i=1}^{K}\sum_{j=1}^{K}C_{ij}}, \end{array}$$

(17) $$\begin{array}{ccc} {\mathrm{Precision}}_{j} & = & \frac{C_{jj}}{\sum_{i=1}^{K}Cij}, \end{array}$$

(18) $$\begin{array}{ccc} Kappa & = & \frac{p_{o}-p_{e}}{1-p_{e}}, \end{array}$$

where

$$\begin{array}{ccc} p_{o} & = & \mathrm{Overall}\;\mathrm{accuracy},\\ p_{e} & = & a^{T}b,\\ a_{j} & = & \frac{\sum_{i=1}^{K}C_{ij}}{\sum_{i=1}^{K}\sum_{j=1}^{K}C_{ij}},\\ b_{i} & = & \frac{\sum_{j=1}^{K}C_{ij}}{\sum_{i=1}^{K}\sum_{j=1}^{K}C_{ij}}. \end{array}$$

The overall accuracy (16) is the number of correct classifications divided by the total number of classified data. The precision (17) is a measure of the accuracy of a specific class *j*. Thus, the precision is the ratio between the number of correctly predicted data of the class *j* divided by the total number of classified data in the class *j*. Cohen’s kappa (18) is a statistical measure of inter-rater agreement or inter-annotator agreement for 2 raters [41]. Cohen’s kappa measures the agreement between two raters who each classify *N* items into *K* mutually exclusive categories, see Table 4 for the interpretation of the values of Kappa index [53]. In Ref. [53] the reader can find more details about the measure of agreement Kappa.

### 4.5. Results and Discussion

We carried out several experiments where we combined the feature spaces described in the Section 4.3. Table 5 gives a brief description about all possible feature space combinations. Column 1 indicates the number of combination and column 2 specifies the feature spaces included in the combination.

We conducted 11 experiments with real data described in Section 4.2. The first three experiments considered only one feature space with the standard GMMF algorithm [29]. The next four experiments were performed using the lowest entropy model [28], see Equations (4) and (5) and the space combinations 4–7 given in Table 5. The last four experiments also used the combinations 4–7, and the segmentation process was conducted using the fusion sources model proposed in this work, see Equations (10)–(12).

Table 6, Table 7 and Table 8 summarize the numerical results of all 11 experiments. These tables present the numerical information about the classification of 5 different crops, see Table 1, the precision of classification for each class, the overall accuracy and Kappa coefficient. We note that the results of the first row of Table 6 and Table 7 differ from those presented in Ref. [28,29]. This is due to, in this results, we only consider sites in the image that correspond to crops in the five categories of interest, i.e., only pixels in the region of interest.

Table 6 shows the results obtained using the standard GMMF algorithm with different features spaces [29,54]. Experiments 1–3 consider the information from Space 1, Space 2 and Space 3 respectively, see Table 5. Note that best result is obtained when using the first three principal components based on all spectral bands, except the TM6 band (Experiment 3). Note also that, the first three principal components using the 10 spectral indexes (Experiment 2) achieved a similar performance to the one obtained with the typical combination of TM2, TM3 and TM4 bands (Experiment 1). This means that for the crop classification using GMMF, the amount of information is relevant.

Table 7 summarizes numerical results for the experiments in which we considered the lowest entropy model [28], see Equations (4) and (5). Experiment 4 combines the Space 1 and the Space 2, Experiment 5 takes into account the Space 1 and the Space 3, Experiment 6 includes Space 2 and Space 3 and Experiment 7 uses all studied spaces. Observe that the combination of different feature spaces together with the lowest entropy model allowed to improve the precision of crop recognition in general, and hence the overall accuracy and Kappa index increased. Table 7 also confirms that not only the information from TM2, TM3 and TM4 (Space 1) is relevant from crop recognition, but also the information derived from other bands.

Table 8 shows the numerical results of the experiments 8–11. These four experiments considered the same feature space combination as in the experiments 4–7 in Table 7, but using the fusion sources model (10)–(12). Note that the Experiment 9 achieved the best performance. This experiment considered the combination of Space 1 and Space 3.

Although the results of the experiments in Table 8 are similar in comparison with the results obtained by means of lowest entropy model, see Table 7, they have a better performance than those obtained by lowest entropy model. In general, the new proposal obtained the best performance compared with our previous conference papers. Furthermore, the study of feature spaces performed in this work also allowed us to improve the performance of our previous work.

Our experimental work corroborated that the vegetation indices enhance the vegetation information, and they are able to distinguish between vegetation and non-vegetation. However, for the classification task, they are not sufficient as an information source to discriminate different types of crops.

Though more information sources we have a greater computation time, for this task we suggest using more feature spaces in order to improve the accuracy of the classification process. For that reason the proposal described in Section 3.2 considers a fusion of multiples feature spaces.

In this work we used feature spaces generated from the pixel information and different spectral bands, however the proposal is more general and it allows to include other feature spaces and not only punctual information, but also local information derived from different image modalities. The study of local information and the fusion of different image modalities is a part of our future work.

On the other hand, Table 9 shows a comparative analysis of different classification methods that have been widely used in the context of crop classification problems. In the experiment we use the real image described in Section 4.2. Here we compare different algorithms implemented in the software MultiSpec, funded by NASA, that is available online [55]. MultiSpec is a freeware multispectral image analysis software developed at Purdue University and the latest release date is on March 2017. In this experiment, we include the following methods: Minimum Euclidean Distance (MED) [56], Maximum Likelihood Classifier (ML) [57], Fisher Linear Likelihood (FLL) [58] and (ESS) [59,60] because they reached the best performance results. We also include two versions of the one vs all multiclass SVM method, the linear and the non-linear Radial Basis Function alternatives, using the winner-takes-all strategy. In this case, we use the matlab built-in function for SVM. In the comparison study we also consider the original version of the probabilistic segmentation approach described in [28,29]. Additionally, for a fair comparison, we include the performance analysis of these algorithms using the best results reached in the feature space study in this work, see Table 6 and Table 7, denoted as MICAI $2014^{*}$ and MICAI $2015^{*}$ in Table 9. Finally, we include the results achieved by our proposal based on the fusion of information sources. Note that for our proposal, and in general for probabilistic segmentation approaches, the natural feature space is the space of probabilistic distributions or the likelihood space, unlike others methods under study where the natural space is the original image. A detailed comparison study of feature spaces and classification methods for crop classification is a very interesting task, however, this is out of the scope of the present research and we leave it for future work.

Based on the comparison results in Table 9, we note that our proposal, in general, outperforms the remainder analyzed methods. The precision, overall accuracy and the Kappa index attained a better performance with respect to our previous conference papers [28,29]. Observe, that the non-linear SVM version with Radial Basis Function leaded to the best classification for class C4, however the best classification for the rest classes was reached by our proposal. On the the hand, the analysis of the feature spaces developed in this work, also allowed to improve the results of the probabilistic methods in [28,29]. The method MICAI $2014^{*}$ corresponds to the GMMF algorithm [29] using the Space 3 studied here. Note that the overall accuracy and Kappa coefficient increased in comparison to the Space 1 used in the original paper in Ref. [29]. Similarly, MICAI $2015^{*}$ refers to the proposal in [28] using the feature space combination Space 1 and Space 3. Note that performance measures of classification also increased.

**Figure 4:**
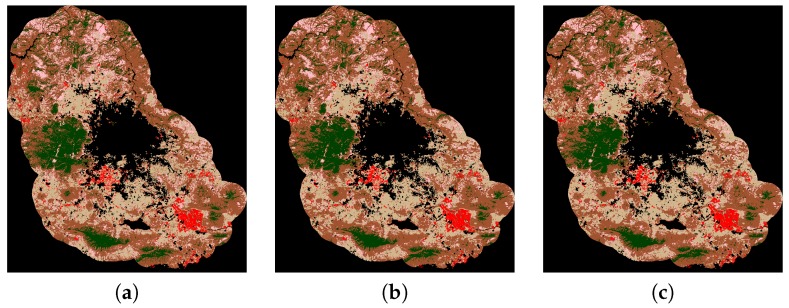
Classification maps of the three most accurate methods given in Table 9: (**a**) non-linear SVM version with Radial Basis Function; (**b**) MICAI $2015^{*}$ and (**c**) Our proposal.

For illustrative purposes, the classification maps of the three most accurate methods given in Table 9 are shown in Figure 4.

## 5. Conclusions

The selection of the feature space is a challenge for any image classification task. Our proposal provides a framework in which different information sources are combined taking into account the entropy of the their probability distribution. To this end, we propose a generalization of the Gauss Markov Measure Field model, in which both, data and regularization terms contain a weight function, that allows to combine different feature spaces, in a such way that information sources with lowest entropy probability distribution have a greater contribution in the classification process. The proposal considers the punctual and contextual spatial information. The weight function of the second term allows us to control the edges between classes obtaining a robust potential based on the likelihoods.

The performance of the proposed approach is evaluated in satellite images for classifying different crops. Although we focused on classification problems in satellite images, the proposal can be used for any image classification task, in which it is necessary to combine several feature spaces, this is a potential research in the future. We also studied different spaces and all possible combinations. The achieved precision indices: overall accuracy and Kappa coefficient demonstrated the good behavior of our approach. Experiments with real images show that the proposed algorithm obtains excellent results compared with algorithms used in the context of satellite image for crop classification. Finally, an interesting problem that emerges from this work is to carry out a detailed comparison study of different features spaces and classification methods for crop classification. We leave this study for future work.

## Figures and Tables

**Table 1:** Vegetation types used for the study.

Class	Vegetation Name
C1	Irrigation agriculture
C2	Temporary agriculture
C3	Forest
C4	Scrub
C5	Pastureland

**Table 2:** Spectral bands of the Landsat-5 TM Sensor.

TM Bands	Wavelength (μ)	Features
TM1	0.45–0.52	B (Blue)
TM2	0.52–0.60	G (Green)
TM3	0.63–0.69	R (Red)
TM4	0.76–0.90	near infrared
TM5	1.55–1.75	mid-infrared
TM6	10.4–12.50	thermal infrared
TM7	2.08–2.35	mid-infrared

**Table 3:** Explored Vegetation indices. In the equations below $\rho_{r}$, $\rho_{g}$, $\rho_{b}$ and $\rho_{NIR}$ denote the reflectance values for the red, blue, green and infrared bands respectively and $\rho_{rb}=\rho_{r}-\gamma \left(\rho_{b}-\rho_{r}\right)$.

Name VI	Formula	References
MSR	$\frac{\frac{\rho_{NIR}}{\rho_{r}}-1}{\sqrt{\frac{\rho_{NIR}}{\rho_{r}}}+1}$	[31]
CI	$\frac{\rho_{NIR}}{\rho_{g}}-1$	[32]
midrule NDVI	$\frac{\rho_{NIR}-\rho_{r}}{\rho_{NIR}+\rho_{r}}$	[15]
GNDVI	$\frac{\rho_{NIR}-\rho_{g}}{\rho_{NIR}+\rho_{g}}$	[16]
EVI	$2.5\left[\frac{\rho_{NIR}-\rho_{r}}{1+\rho_{NIR}+6\rho_{r}-7.5\left(\rho_{b}\right)}\right]$	[33]
SARVI	$\frac{\left(1+L\right)\left(\rho_{NIR}-\rho_{rb}\right)}{\left(\rho_{NIR}+R_{rb}+L\right)}$	[34]
RDVI	$\frac{\rho_{NIR}-\rho_{r}}{\sqrt{\rho_{NIR}+\rho_{r}}}$	[31]
SAVI	$\frac{\left(1+L\right)\left(\rho_{NIR}-\rho_{r}\right)}{\rho_{NIR}+\rho_{r}+L}$	[35]
MSAVI	$\frac{1}{2}[(2\rho_{NIR}+1-\sqrt{{\left(2\rho_{NIR}+1\right)}^{2}-8\left(\rho_{NIR}-\rho_{r}\right)})]$	[36]
WDRVI	$\frac{\alpha \times \rho_{NIR}-\rho_{r}}{\alpha \times \rho_{NIR}+\rho_{r}}$	[37]

**Table 4:** Interpretation of Cohen’s kappa measure.

Cohen’s Kappa	Interpretation
<0	Poor agreement
0.00–0.20	Slight agreement
0.21–0.40	Fair agreement
0.41–0.60	Moderate agreement
0.61–0.80	Substantial agreement
0.81–1.00	Almost perfect agreement

**Table 5:** All possible combinations of feature spaces.

N. Combination	Feature Space Combination
1	Space 1
2	Space 2
**3**	**Space 3**
4	Space 1 + Space 2
**5**	**Space 1** + **Space 3**
6	Space 2 + Space 3
7	Space 1 + Space 2 + Space 3

**Table 6:** Numerical results of experiments using the GMMF model with only one feature space.

Experiment	Precision					Overall Accuracy	Cohen’s Kappa
	C1	C2	C3	C4	C5		
1	0.86	0.83	0.89	0.85	0.63	0.8331	0.7520
2	0.84	0.81	0.89	0.87	0.62	0.8324	0.7528
**3**	**0.83**	**0.82**	**0.91**	**0.89**	**0.65**	**0.8506**	**0.7801**

**Table 7:** Numerical results of segmentation experiments using the modified GMMF with minimum entropy criteria.

Experiment	Precision					Overall Accuracy	Cohen’s Kappa
	C1	C2	C3	C4	C5		
4	0.86	0.83	0.92	0.87	0.67	0.8472	0.7726
**5**	**0.89**	**0.84**	**0.94**	**0.88**	**0.70**	**0.8624**	**0.7955**
6	0.85	0.83	0.91	0.89	0.64	0.8485	0.7770
7	0.87	0.84	0.93	0.88	0.67	0.8588	0.7904

**Table 8:** Numerical results of experiments with the GMMF model of fusion of different sources with μ=1.

Experiment	Precision					Overall Accuracy	Cohen’s Kappa
	C1	C2	C3	C4	C5		
8	0.88	0.83	0.92	0.87	0.67	0.8497	0.7767
**9**	**0.90**	**0.84**	**0.94**	**0.89**	**0.71**	**0.8649**	**0.7993**
10	0.88	0.82	0.91	0.89	0.66	0.8517	0.7812
11	0.90	0.83	0.93	0.89	0.69	0.8600	0.7923

**Table 9:** Numerical results of different classification methods. MICAI $2014^{*}$ and MICAI $2015^{*}$ denote the best results of the proposals in [28,29] under the feature space study conducted in this work.

Method	Feature Space	C1	C2	C3	C4	C5	Overall Accuracy	Kappa
MED [61]	Landsat-5 TM	0.82	0.73	0.54	0.80	0.29	0.6244	0.4781
ML [62]	Landsat-5 TM	0.65	0.73	0.72	0.76	0.40	0.6928	0.5558
FLL [63]	Landsat-5 TM	0.88	0.74	0.71	0.75	0.46	0.7105	0.5743
ESS [60]	Landsat-5 TM	0.76	0.70	0.80	0.74	0.56	0.7257	0.5811
SVM linear	Landsat-5 TM	0.77	0.74	**0.96**	0.73	0.19	0.7498	0.6071
SVM rbf	Landsat-5 TM	0.79	0.83	0.90	**0.90**	0.66	0.8536	0.7859
MICAI 2014 [29]	Space 1	0.86	0.83	0.89	0.85	0.63	0.8331	0.7520
MICAI $2014^{*}$	Space 3	0.83	0.82	0.91	0.89	0.65	0.8506	0.7801
MICAI 2015 [28]	Spaces 1 & 2	0.86	0.83	0.92	0.87	0.67	0.8472	0.7726
MICAI $2015^{*}$	Spaces 1 & 3	0.89	**0.84**	0.94	0.88	0.70	0.8624	0.7955
Proposal	Spaces 1 & 3	**0.90**	**0.84**	0.94	0.89	**0.71**	**0.8649**	**0.7993**
